# LOCAL KNOWLEDGE OF ADAPTIVE STRATEGIES AGAINST MALARIA ENDEMICITY IN THE OKAVANGO DELTA, BOTSWANA

**DOI:** 10.21010/Ajid.v16i2.3

**Published:** 2022-05-06

**Authors:** Maphane Dirontsho, Ngwenya Barbara Ntombi, Kolawole Oluwatoyin Dare, Motsholapheko Moseki Ronald, Pagiwa Vincent

**Affiliations:** * Okavango Research Institute, University of Botswana, Private Bag 285, Maun, Botswana; # Office of District Commissioner, Ministry of Local Government, Gaborone, Botswana

**Keywords:** local knowledge, adaptation, Botswana, malaria control, Okavango Delta

## Abstract

**Background:**

An increasing recognition of the need to eliminate malaria infection globally and particularly in Africa where more than 90% of the cases originate, should enhance community awareness and participation in the same. The perennial freshwater of Okavango Delta located in northern Botswana, which is a source of rural livelihoods and a suitable habitat for the malaria-causing mosquito, and where malaria is endemic provides a suitable environment for the study. Therefore, local households’ adaptive strategies against malaria transmission in the Okavango Delta were investigated.

**Materials and Methods:**

The data used in this paper is a subset or retrospective cohort of 79 households that reported malaria cases/incidences during the first community level household survey conducted from October-November 2015 on 355 households in Shakawe and Ngarange. The households were selected through stratified random sampling in the two study villages. The retrospective cohort household survey was conducted from 7-19 August 2016, in the two study sites. Data were collected through socio-economic survey, key informants’ interviews and focus group discussions.

**Results:**

The results obtained indicate that most cohort households embraced several adaptive strategies against malaria transmission. These included, *inter alia*, knowledge capacity building through attendance of health information and education workshops (69%), modifications of houses structures (49.4%), timing of activities and restricting movement at certain times of the day (43%).

**Discussion:**

Although most of the stated adaptive strategies such as house screening were not exclusively aimed towards malaria prevention and adaptation, they nonetheless had postive spill over effect that could be a catalyst for eliminating malaria in the study area.

## Introduction

Malaria elimination is a worldwide, topical health issue. Globally, there are over 200 million cases and 600 000 deaths occurring annually from malaria infection. More than 90% of the cases originate from Africa (WHO, 2013). It has been observed that disease control interventions have significantly reduced the burden of local malaria transmission in many countries. Factors contributing to sustained malaria decline in some areas and the apparent failure in other epidemiological settings could be as a result of the use or non-use of novel insecticides, respectively (Chiyaka *et al.*, 2013). This implies that there might have been other factors contributing to malaria transmission. Nonetheless, scaling up prevention and treatment interventions supported by the Roll Back Malaria Partnership has significantly decreased malaria burden in parts of sub-Saharan Africa (Chiyaka *et al.*, 2013). Compared to the rest of the African continent, the implementation of malaria control programme has been successful in southern Africa. However, malaria is still a health problem in the region (Mabaso *et, al*., 2007). In Mozambique, for instance, transmission remains the highest during the wet season (Abellana *et al.*, 2008). It is noteworthy that almost 37% of the 2 065 398 inhabitants of Botswana is at risk of malaria (Kgoroebutswe *et al.*, 2020).

Malaria transmission in Botswana peaks during the rainy season, generally between November and April (Chirebvu *et al.*, 2016). The recently adopted country strategy is toward malaria elimination engendered by an overall decline in malaria transmission from 4.2% of the total population in 2000 to 1% in 2008 (Simon *et al.*, 2013). Vector characterization surveys conducted in 2006 revealed that *Anopheles arabiensis* is present in all malaria areas of Botswana (Simon *et al.*, 2013). However, *Plasmodium* *falciparum* parasite species is responsible for most of the deaths recorded from malaria (Chirebvu *et al.*, 2016).

Although annual Indoor Residual Spraying (IRS) and door-to-door distribution of long-lasting insecticidal nets (LLINs) has reduced malaria related fatalities in the country, there are still specific malaria ‘hot spots’ in Botswana, particularly in the Okavango sub-region. From 2009 to 2012, the region accounted for 22% of total cases of malaria reported in the country (Simon *et al.*, 2013). The country’s profile in relation to malaria infection is designated as epidemic while parts of the Ngami and Okavango Delta are endemic. This is primarily attributed to the presence of swamps within the Delta area that provide breeding sites for malaria vectors (MoHW, 2009).

Arguably, local practices designed to mitigate impacts of any given endemic environmental hazards are complex since adaptive responses often evolve over historical times and space across generations through trial and error (Ngwenya *et al.*, 2016). The environment in which individuals and populations live has a dominant effect on their health. The people have, over the years, gained an understanding of the diverse aspects of the environment through experience and knowledge, which have been passed from generation to generation, thus ensuring community survival (Ngwenya *et al.*, 2016). This is the basis for local knowledge, which enables them to make decisions in the fields of agriculture, healthcare, natural resource management and other activities (Ngwenya *et al.*, 2016).

### Environment/ecological issues and malaria endemicity in the Okavango Delta

People affected by disasters often play a crucial role in taking preventive measures to protect themselves from the impacts (Batterbury and Forsyth, 1999). Local knowledge on environmental dynamics such as floods, rainfall and temperatures enable people to understand environmental-related health problems such as malaria and how to prevent, treat and adapt to them. Indeed, ecology significantly impacts on the kind of knowledge and innovations that are developed in a given specific context (Kolawole, 2015). Local knowledge in environmental changes is thus utilised to identify vulnerability to malaria and facilitate adaptive responses to the disease (Vedwan, 2006).

In this article, local knowledge of malaria prevention and adaptation is defined as a system of concepts, practices, beliefs and perceptions that people utilise to prevent, reduce or avert malaria transmission in the malaria endemic villages along the fringes of the Okavango Delta area in north-western Botswana. This includes the way people in the area observe and utilise knowledge and practices accumulated over time to adapt to changes in their surroundings in order to reduce vulnerability to disease transmission; the process involve how they perceive risk and solve problems; and how they access services and validate information (Vedwan, 2006).

Adaptation to malaria endemicity should, therefore, build upon local knowledge as the adaptive capacity of the affected community. It should be recognized that the enhancement, sharing and incorporation of indigenous knowledge in compliance with the ethical principles is critical for ensuring effective and locally appropriate adaptation. Adaptation to malaria endemicity planning must, therefore, consider historical experience and local knowledge developed by the local communities to address disease transmission trend and prevalence in the past, and their perspectives on the knowledge of shocks and vulnerability (Chikaire and Nnadi, 2011).

Local knowledge about malaria prevention and treatment is accumulated by communities over time and, therefore, becomes encoded as facts and information relevant to a specific locale and socio-cultural and environmental context. Local knowledge can also include specific skills or experiences acquired in a location (see Kolawole, 2015; Srinivas, 2015). In this regard, local knowledge can be understood as a body of knowledge, which communities draw upon to address certain problems, including malaria. Used as a local adaptation tool used to address pre-existing conditions such as malaria endemicity, local knowledge helps experts or scientists to reach a consensus with local actors during the implementation of intervention programs in the affected community (Srinivas, 2015). Local knowledge in health issues encompasses both generalised and specialised prevention, and treatment competencies at individual, group and community levels.

Communities living in malaria endemic areas have over the years developed appropriate concepts relating to malaria treatment and prevention. These concepts are based on traditional notions of health and disease and are derived from observations of the connection between environmental conditions, vectors and malaria (Kipsisey, 2008). Local communities uphold the principle of prevention through practices such as using traditional medicines (herb or animal-based etc.) and avoiding certain environments (United Nations, 2015). Their use of local knowledge of malaria prevention has engendered self-treatment at home without visiting health care facilities (Toé *et al.*, 2009). Using different local preventative measures or a combination of such with scientific techniques is a commonplace in these communities, depending on how these measures work best for them. In this context, local knowledge could enhance modern techniques and prevent reintroduction of post-elimination interventions (Toé *et al.*, 2009).

In Tanzania, Gessler *et al*. (1995) suggest that traditional healers have specialized knowledge of treating malaria with herbs. Several traditional approaches of controlling malaria are known and have been practised by communities living in malaria endemic zones. None of these approaches have been investigated and this raises serious sustainability questions on their effectiveness in malaria control (Waako *et al.*, 2010). Currently systematic research on local knowledge in southern Africa is very limited. There is, therefore, the need to conduct systematic research to identify and investigate how local knowledge could be infused in policy/program interventions in health and other sectors in order to optimize sustainably, positive outcomes (Kolawole, 2015; Dunn, 2011). More importantly, harnessing local knowledge is likely to garner active participation of communities in malaria elimination activities (Whittaker and Smith, 2015). In Botswana, there is a gap in how households living in malaria endemic communities of the Okavango Delta harness local knowledge to prevent and or enhance adaptation against susceptibility to malaria transmission. By identifying and analysing household adaptive strategies against malaria transmission in the Okavango Delta, Botswana, this paper intends to partly fill this knowledge gap.

### Study Area

The Okavango River originates from the upland plains of Angola, forming the Okavango Delta and the Chobe/Linyanti rivers in Botswana. The two river systems provide the major perennial surface water and constitute 95% of the country’s water (Government of Botswana, 2001). The Okavango Delta is in the terminal section of the Okavango River Basin. The main tributaries of the Okavango River are the Quito and Cubango Rivers situated in central Angola, which are catchments that receive rainfall of 876 and 983 mm/year, respectively (McCarthy *et al.*, 2000). Floods increase the transmission of communicable and non-communicable diseases through increased number and range of vector habitats. The annual flood pulse, which is fed by the flood waters from the Angolan highlands inundates an area that varies in terms of coverage from 5 000km^2^ to 120 000 km^2^ from one year to another (Wolski, 2006). The floods are usually experienced between April and June and thereafter recede due to evapo-transpiration and infiltration (McCarthy *et al.*, 2000). The panhandle of the delta is about 100 kilometres and then divides into several fan-like distributaries comprising interconnected channels and floodplains interspersed with a series of islands of various sizes (McCarthy *et al.*, 2000; Wolski, 2006).

One of the ecological components created by an ecosystem characteristic of the Okavango Delta is the floodplains. Rainfall is largely responsible for creating conditions that allow enough water for mosquito breeding sites and is therefore recognized as one of the major factors influencing malaria transmission. Botswana experienced a very high malaria season in 1993 and this was associated with an above average rainfall of the 1992-1993 season following the 1991-1992 devastating drought (Thomson *et al.*, 2005). The resources of the Okavango Delta play a major role in sustaining the livelihoods of the local people; the annual floods provide opportunities for water-related activities such as flood recession farming (*molapo* farming), which results in conducive habitats for the malaria vector. Like any other wetlands, the Delta also provides habitats for a diverse range of water-dependent pests, pathogens and vector species.

Mosquitoes, including species which transmit pathogens such as malaria parasites, thrive in such ecologically diverse environments.

Two study sites situated along the Okavango Delta panhandle, namely Shakawe and Ngarange villages, were selected. The selection criteria included *inter alia*, all year-round malaria transmission and high incidence of malaria cases, which are consecutively recorded by the Ministry of Health and Wellness (MoHW, 2014), proximity to the perennial surface water; exposures to periodic flooding episodes hence vulnerability to contracting the disease, and community access to high and low order health facilities. Shakawe has a clinic with a maternity wing while Ngarange has a health post.

**Figure 1:**
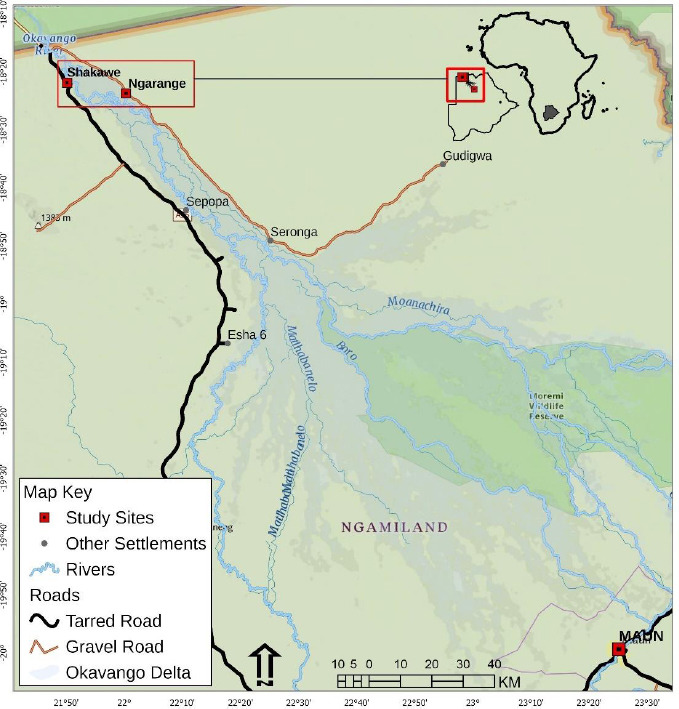
Map of the study area showing the Shakawe and Ngarange study sites in the Okavango Delta area (Courtesy of Anastacia Makati, ORI GIS Lab).

Shakawe (Figure 1) is the largest fishing village in Botswana (Mmopelwa, 2005). This livelihood activity has been documented as an avenue through which exposure to malaria vectors occurs (Barai *et al.*, 1982). Shakawe has a population of 6 693 (CSO, 2011) and the village, which is growing steadily, has a shopping centre, government offices, NGOs and several private safari lodges. Ngarange village is on the eastern part of the panhandle along the Okavango River (Figure 1). It has a population of 988 people (CSO, 2011). Livelihood activities in both villages include but not limited to fishing, gathering of wild and aquatic fruits, pastoral and arable molapo farming. The main ethnic groups found in these villages are the HamBukushu, Baherero, Bayeyi, Basarwa and Batawana. The livelihoods of the people particularly the HamBukushu and Bayeyi are traditionally dependent on water resources (such as fish). The Bayei are also traditionally dependent on *molapo* farming.

## Methods and materials

### Research design

Using an exploratory, cross-sectional and analytical research designs, this study adopted a mixed methods approach to obtain both quantitative and qualitative data. The approach is usually used when a research method (whether qualitative or quantitative) is not adequate to address the research problem (Klassen *et al.*, 2012). Mixed methods is used for complementarity, expansion and explanation purposes (Klassen *et al.*, 2012). While the quantitative aspect of this study addresses the household survey component, which seeks to determine the relationships between variables, the qualitative aspect explores the experiences, attitudes and perceptions of study participants and elucidate on the development and operation of their knowledge and ideas at the local level (Kitzinger, 1995).

### Sampling

### Initial sampling

A stratified sampling procedure was used to select the study participants. First, the village wards (known as *dikgotla*) were stratified based on size (small, medium, large) in both villages. Second, records of malaria prevalence in each ward were collected from the local clinic in Shakawe. These cases were also used to determine whether a ward has low, medium or high prevalence of malaria infection. The sampling frame was derived from a household listing for selected wards (strata) in the two villages. Seven (7) out of the fourteen (14) wards in Shakawe and all the six (6) wards in Ngarange were selected because there was no significant variation in ward size, and malaria cases were sparsely distributed across the wards making it difficult to classify them as high or low. Simple random selection of households proportional to size of the ward (in terms of household number) was then carried out. The sample size was determined from the total number of listed households in each village where 877 households were recorded in Shakawe and 300 in Ngarange. The confidence level was fixed at 95% with a margin error of plus/minus five (^+^5). A total of 274 households from Shakawe and 81 from Ngarange, making a total of three hundred and fifty-five (355) households were, therefore, sampled and interviewed.

### Cohort study sampling

A retrospective cohort study type uses data, which were already collected for other purposes (Sedgwick, 2014). Such study type, which requires previously collected data on exposure status for both cases and non-cases (Sedgwick, 2014) tends to be prone to selection bias due to maturation of study participants and or failure to follow up on some of the respondents. Losses of study participants, however, do not necessarily nullify the study. This paper presents results derived retrospectively from a cohort in which 355 households previously used for the socio-economic survey, were used as a sampling frame in order to do a follow-up on respondents who had experienced malaria. A total of 129 households with malaria cases were initially identified from the main socio-economic survey of 2015. However, the household interviews were eventually conducted only in fifty-four (54) households in Shakawe and twenty-five (25) in Ngarange, totalling seventy-nine (79) households. The initial sample size could not be achieved because of (i) the absence of targeted household members from their homes; (ii) participant loss due to relocation or death; and (iii) some participants’ unwillingness to participate, presumably denying that they never had a malaria patient in their households.

### Criterion-based purposive sampling

Criterion-based sampling is used to identify and select all cases that satisfy a pre-set criterion of importance (Palinkas *et al.*, 2015). This sampling technique was used with consideration for personal skills, age, leadership position in the community, and occupation/profession. The key informants were viewed as either custodian of local knowledge or opinion leaders able to make inferences about utilization of local knowledge on malaria epidemic in their area. A total of sixteen (16) key informants (10 from Shakawe and 6 from Ngarange) were purposively sampled and interviewed.

### Focus group discussions (FGDs)

FGDs served to explore the meanings of survey findings that cannot be explained statistically. It helps to obtain a range of opinions/views on a topic of interest and to collect a wide variety of local terms (Parsons & Greenwood, 2000). One FGD was conducted in each study site. Criterion-based purposive sampling was also used to select the FGD participants. The selection criteria for FGDs were age, gender, length of residency, and household livelihood activities. Some of the FGD participants had been identified much earlier during the household interviews.

### Data Collection

Permission to conduct this study was earlier obtained from the Botswana Ministry of Health, Ngamiland District Health Management Team (NDHMT) Permit No: M 6/50/12 I). To ensure the validity and reliability of the instruments, a pre-tested set of data collection tools were used to obtain both quantitative and qualitative information from the study participants. Data on household socio-economic background, sources of livelihood, local knowledge utilization on malaria prevention, local adaptive strategies against malaria and perceptions on health institutions were collected. In assessing the adaptive strategies against malaria transmission, respondents were required to answer ‘Yes’ or ‘No’ to a set of 11 adaptive strategies, which were compiled from literature review and previous socio-economic survey results. Placed on a 5-point Likert rating scale, a set of items/statements were presented to the respondents who were asked to rate how often they used the strategies. They were also requested to rate the effectiveness of the strategies through another set of items placed on a 5-point rating scale. The frequency of use was rated as always (1 point); usually (2 points); Sometimes (3 points); Seldom (4 points); and Never (5 points). Effectiveness was measured on a rating scale of very high (1 point), high (2 points), medium (3 points), low (4 points) and very low (5 points).

Key informants in Shakawe and Ngarange were interviewed using a semi-structured interview guide with both semi-closed and open-ended questions. The informants included chief/headman, health officers and village development committee chairpersons, malaria focal person, community health nurses, medical doctors, district health management team members, traditional healers, elderly community members and traditional healers. A key informant guide was used to collect data on: (a) adaptive strategies against malaria; (b.) utilisation and adherence to interventions; and (c.) awareness of mosquito and malaria patterns.

Secondary data sources were also consulted to supplement and strengthen the validity of primary data. This included desk review from unpublished reports, journals, online databases, books, government publications and the Internet. Secondary data was used to capture past changes and developments on malaria infection and its trends over the years.

### Data processing and analysis

Quantitative data quality assurance process was done in the field to check and correct data capture errors. The data were coded, entered and cleaned in SPSS spread sheet. The coding process involved categorisation and allocation of numerical codes to all close-ended responses. Data were summarised by using descriptive statistics (such as frequency and percentages), measures of central tendency (i.e., mean), and charts.

Qualitative data were analysed using content analysis procedure described by Mayring (2004). The process entails ‘structuring’ data with the intention to filter the relevant content and pre-determined categories (themes). All the categories were reviewed, and others merged to form sub-categories. Content analysis process requires reading and re-reading data sources (key informants and FGD transcripts (Mayring, 2004)

## Results

### Socio-demographic background

Analysis shows that 79 households sampled from the cohort study comprised 32.9% males and 67.1% females and having an age range of 18 to 90 years. The respondents’ average age was 38 years. Majority (43%) of the respondents were of the age group 29-39 years. The household types comprised male-headed (55.7%), *de fact*o female-headed (38%), and *de jure* female-headed (6.3%) households. The highest level of education attained in most households was the Senior Secondary level (43.2%) and only 1.3% of respondents attained primary school education.

### House-hold adaptive strategies

The results indicate that the Okavango Delta communities adapted to several strategies to guard or protect against malaria transmission. The strategies observed were categorised into the following classes/types: a) structural, which included modifications on houses to help limit and wade off mosquitoes from homes and screening doors and windows/ installation of gauzes to block vector entry; b) timing activities strategy, which includes partaking in activities at certain times that present less exposure to mosquito bites during the day, restriction of movements when mosquito biting chances are high; and c) other strategies, which included having livestock in close proximity as alternative hosts to reduce biting rate on the humans, livelihood diversification and attending informative health workshops for capacity building against malaria. The description of each strategy in terms of their pattern and frequency of utilisation as well as its perceived effectiveness are provided below:

### Structural strategies

**Figure 2:**
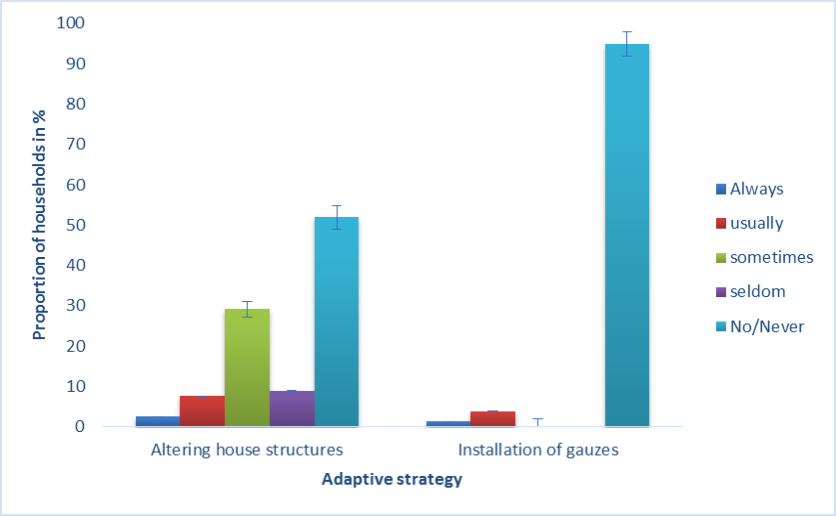
A graph depicting utilisation of structural adaptive strategies in mosquito bite prevention by households in the Okavango Delta area

Structural strategy entailed any modification or change in accommodation facility to guard against mosquito invasion. Figure 2 below shows that altering house structures was the most embraced adaptive strategy. Forty-eight percent (48%) of the respondents altered their house structures. While 29% of them sometimes altered their houses, 9% seldom altered their house structures. Also, while 7.6% of them would usually alter their house structures, only 2.5% of the respondents always did so. The second most adopted strategy was construction of modern houses, utilized by 36.7% of the households. This construction of modern houses as an adaptation strategy is not shown in Figure 2 because the frequency of use (as measured on the Likert scale responses) is a misnomer for measuring this strategy.

**Figure 3:**
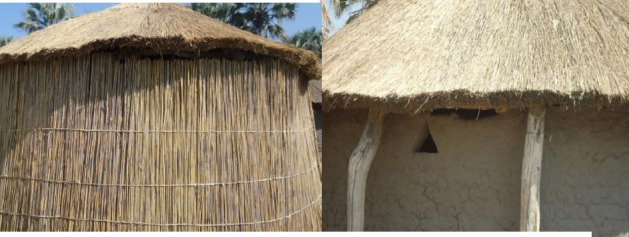
Picture showing eaves in reed and mud hut when used as structural adaptive strategies in mosquito bite prevention by households in the Okavango Delta area (Courtesy: Barbara Ngwenya)

**Figure 4:**
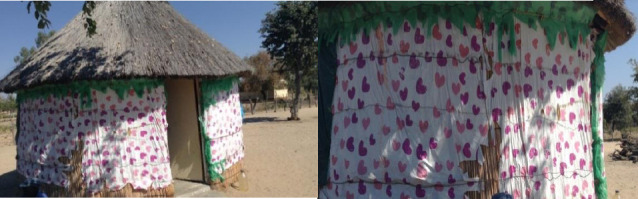
Pictures showing houses screened with plastic material as a structural adaptive strategy in Shakawe (Courtesy:Dirontsho Maphane)

**Figure 5:**
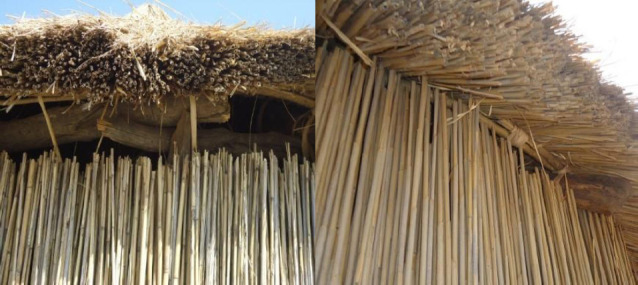
Pictures showing eaves in the reeds when used as in structural adaptive strategy in mosquito bite prevention (Courtesy: Barbara Ngwenya)

Field observations and informal discussions suggest that, even though architecture structural change strategies were not very evident throughout the villages, there were modern houses (with brick walls, corrugated iron roof and windows) in almost every interviewed and non-interviewed household. However, it was also evident that newly constructed reed houses and some mud houses had eaves that could allow mosquitos in and out at dusk and dawn (Figure 3). Observational evidence (as shown in Figure 4) indicates that one of the informants used house screening (that is, using plastic to wrap the walls of the reed hut) in order to wade off mosquitoes from entering and exiting the hut through the spaces between the reeds and thatched (grass) roof as would be the case in an unsealed structure (Figure 5).

### Timing of activities

**Figure 6:**
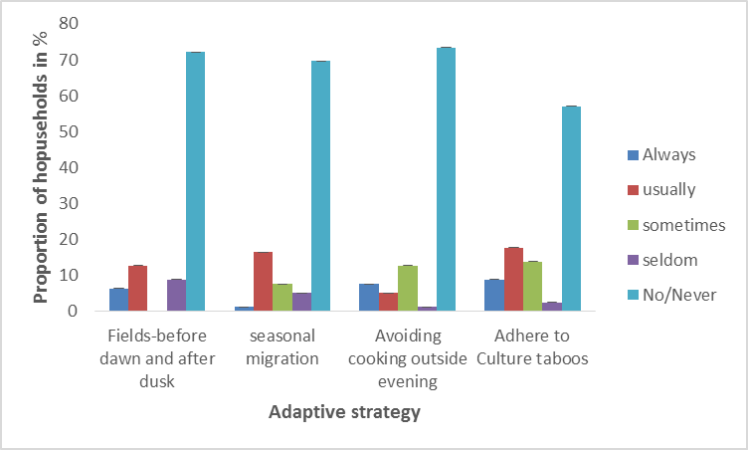
Utilisation of timing of activities as adaptive strategy in mosquito control by households

This could also include avoiding doing activities at the peak of mosquito bite; adaptive strategy involved carrying out activities at some particular time of the day or season when the chances of mosquito bites are low as compared to any other time of the day . Figure 6 below shows that majority (43%) of the households adhered to taboos that restrict movement at certain times of the day. While approximately 9% of them always adhered to taboos restricting movement, about 17.7% of them usually did so. Nonetheless, about 14% of the respondents sometimes adhered to taboos while 2.5% of them seldom adhered.

Seasonal commuting to and from the field and cattle post was the second most embraced strategy (30.4%) in the timing of activities. While 16.5% of the households usually engage in this back and forth commuting, 7.6% of them sometimes commuted, 5.1% seldom and 1.3% always engaed in commuting. Tending to crops after dawn and before dusk (27.8%) and avoiding out-door cooking in the evening (26.6%) were the least adopted strategies.

### Other strategies

Other adaptive strategies included diverting mosquitoes away from the human host, through construction of livestock kraals close to homesteads; capacity building through information sharing at health workshops; and diversifying livelihood activities with high risk of malaria transmission to less risk activities such as formal employment.

**Figure 7:**
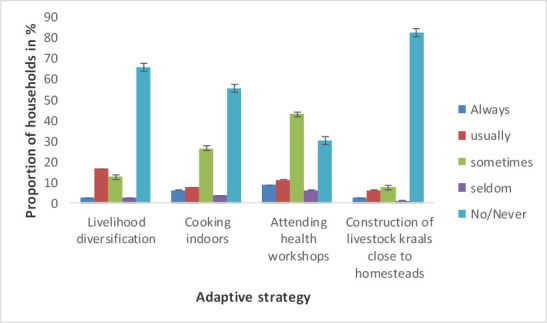
Utilisation of other adaptive strategies by households

Figure 7 shows that attending health workshops is the most frequently used (69.6%), across all levels of use, followed by cooking indoors (43%), and livelihood diversification (35.4%). Nonetheless, construction of livestock kraals in close proximity to homesteads is the least adopted (17.7%). Raising awareness during community gatherings such as at Kgotla meetings has been the most observed strategy for coping with and adapting to malaria situation in the study area. Almost 38% the key informants mentioned attendance of health workshops and kgotla meetings as some of the various platforms used by health workers to raise awareness in the community. As everyone virtually utilizes services at the clinic, weekly Information Education and Communication (IEC) events by the health staff at the clinic was also mentioned by 25% of the informants as other means of adaptation to malaria. Some of the key informants (including community informants and a health worker) remarked thus:

“We are enlightened very much through participation in community as well as at the Kgotla meetings and the messages reach us through the healthcare workers” (Community key informant).

“Healthcare workers teach us every week at the clinic, especially that most of us go to the clinic in large numbers when we are in need of health services; everyone, adults and children, men and women” (Community key informant).

“We raise awareness of different health issues including malaria prevention during weekly IEC at the clinic. Community members are also involved in the fight against malaria through attendance of our occasional health workshops where different community members including village health committee, religious leaders and traditional healers attend. This also takes place during the attendance of kgotla meetings” (Community healthcare worker).

### Effectiveness of adaptive strategies

Households were asked to rate the effectiveness of each of the strategies they identified. Table 1 indicates that altering house structures was perceived as the most effective structural strategy and was rated very high and high by 15.8% and 31.6% of the respondents, respectively, as compared to construction of modern houses, which was rated very high (17.2%) and high (34.5%) by the households. Also, installation of gauzes was rated very high (25%) and high (75%) by the households. In terms of timing of activities, tending to crop in the fields before dawn and after dusk was perceived to be the most effective strategy, and was rated highly effective and highly effective by 40.9% and 31.8% of the households, respectively. Movement restriction at certain times of the day, which is enforced through adherence to cultural taboos was rated by 28.6% of the households as very highly effective. Among the category of ‘other strategies’ identified by the households, attending health workshops was found to be the most effective, and was rated as having very high (32.7%) and high (41.8%) level of effectiveness.

**Table 1:** households’ rating of adaptive strategies’ effectiveness

Strategy	Very high	high	Effectiveness in % moderate	low	Very low
1. **Structural**					
Altering house structures	15.8	31.6	34.2	7.9	10.5
Installation of gauzes	25	75	0	0	0
Construction of modern houses	17.2	34.5	31	10.3	6.9
2. **Timing activities**					
Fields before dawn and after dusk	40.9	31.8	27.3	0	0
Seasonal migration	4.2	12.5	20.8	50	12.5
Avoiding cooking outside evening	19	38.1	33.3	9.5	0
Adhere to Culture taboos	28.6	34.3	22.9	5.7	8.6
3. **Others**					
Attending health workshops	32.7	41.8	21.8	3.6	0
Livelihood diversification	3.7	18.5	55.6	11.1	11.1
Cooking indoors	28.6	25.7	25.7	5.7	14.3
Construction of livestock kraals close to homesteads	21.4	14.3	14.3	21.4	28.6

**Source**: Household survey (2015)

The respondents opined that the community was aware of the symptoms and seasonal outbreak of malaria through workshops and IEC. Also, they indicated that the response to and acceptance of government interventions against malaria also plays a major role in protecting people against the disease and must, therefore, be considered as other adaptive strategies. Livelihood diversification and construction of kraals close to homesteads were the least effective in this category as shown in Table 1 above. Most households rated livelihood diversification as moderate in terms of its effectiveness. It is noteworthy that cooking indoors had the lowest rating on the scale of effectiveness.

## Discussion

The results obtained indicate that the most effective adaptive structural strategy against malaria transmission was the alteration of the structure of the traditional mud huts. This strategy is the most affordable because the building materials are readily available. Indeed, a simple modification of a typical rural house design is a relatively low-cost technique for wading off mosquitoes and by so doing reducing the chances of malaria transmission. Given that house structure modification was reported to be underexploited in malaria control in other parts of sub-Saharan Africa (SSA) (Tusting *et al.*, 2015), this strategy is conceived as a major stride in malaria prevention for households in the Okavango Delta. Improving house structures may be beneficial particularly in the SSA where up to 80-100% of malaria transmission occurs indoors at night (Huho *et al.*, 2013).

Closing the gap between the mud wall and grass roofing at the construction stage is the commonest structural change usually adopted in the Okavango Delta. This reduces the chances of mosquitoes entering the house to a certain extent. Generally in the SSA, traditional homes are considered to have mud walls, thatched roofs and earth floors, open eaves, with neither ceiling nor screening (Bradley *et al.*, 2013). Closed eaves have been associated with less likelihood of malaria infection and clinical malaria in Uganda (Tusting *et al.*, 2015). This modification is likely to alleviate malaria incidences particularly in the SSA where the primary African vector (*Anopheles gambiae*) has been observed to find hosts by following odour trails close to the ground and flying upwards, upon encounter with vertical surface (Tusting *et al.*, 2015). This behaviour has also been observed in *Anopheles arabiensis* (the main malaria vector in Botswana), which enters houses through open eaves, after flying upwards due to encounter with wall surfaces. Therefore, the households in the study area can benefit from investing in housing improvement to block the entry routes of malaria vectors (Lindsay *et al.*, 2002).

The second most used structural strategy against malaria transmission was the construction of modern houses. Modern houses with brick walls and corrugated iron roofs were found to be associated with lower mosquito biting rate compared to the traditional mud and reed hut (Ondiba *et al.*, 2018; Mburu *et al.*, 2018). This finding is similar to that of Tusting et al. (2015) in which modern wall materials were found to be associated with a reduction in the chances of malaria infection, and modern roof materials were associated with up to a two thirds reduction in the incidence of clinical malaria. In Tanzania, houses with brick walls and corrugated iron roofs are associated with lower levels of malaria-associated anaemia compared to poorly constructed mud-wall houses (Kahigwa *et al.*, 2002). Similar observations were made in malaria endemic communities in Sri Lanka where the risk of getting malaria was greater for inhabitants of uncompleted houses, with thatched roofs and walls made of mud-woven coconut palm leaves, compared to better-constructed houses with complete brick and plastered walls, and tiled roofs (Atieli *et al.*, 2009).

The reduction of the entry of malaria vectors into houses through improved house construction has long been recommended as a way of reducing human exposure to mosquito bites and subsequent malaria infection (Lindsay *et al*., 2002). House screening has contributed to the achievement of malaria elimination in many countries worldwide (Lindsay *et al.*, 2002). It was the first intervention near Rome in Italy after the link between malaria and mosquitoes was established (Ferroni, Jefferson and Gachelin, 2012). It was then later shown to reduce malaria risk in India and South Africa (Tusting et al., 2015; Keiser et all., 2005). However, findings showed that this strategy is the least adopted by the Okavango Delta communities indicating that the benefits of house screening/installation of gauzes for protection against mosquito bites have not yet been recognised in the area, perhaps because of local people’s financial limitations.

Seasonal migration is a socio-cultural practice interplaying and impacting on adaptation to malaria endemicity. However, it is not clear how much seasonal migration contributes to reduction or increase in malaria cases. Findings, however, reveal that some of the households migrated seasonally mainly for agricultural purpose. This may possibly contribute to malaria cases, depending on the level of transmission risk at the farms (cattle post/fields) in the study area. For example, seasonal migration contributed to the malaria cases in the south of Azerbaijan in Asia where 40% of people migrate during the agricultural season (Grambsch and Menne, 2003). However, population movements usually and seriously hamper vector control activities (Service, 1989**)**. The efforts to locate and spray temporary shelters of the seasonally migrating tribal groups in Bihar Province in India have been hampered by their settlements within dense forests, in brush wood huts and their inability to inform anyone about their movements (Service, 1989). Although the Okavango Delta respondents pointed out that their farmstead structures were not suitable for the IRS, they, however, indicated that the IRS teams were not reaching their shelters in the arable fields and cattle-posts.

This study also showed that the chances of mosquito bites are reduced when people visited their farms after dawn and before dusk. Avoiding outdoor cooking in the evening was also perceived to lessen the chances of mosquito bites. These strategies are more likely to function in avoiding bites from the *exophagic* (outdoor feeding) and *exophilic* (outdoor resting) vector species, including the *Anopheles arabiensis*, which is the commonest in Botswana. Some vector mosquitoes, particularly the Anopheles are most active in twilight periods at dawn and dusk or in the evening, therefore making malaria transmission to occur mostly during these times (Mboera *et al.*, 2013). Given this scenario, it has been observed that exposure to mosquito bites can be minimized by modifying patterns of activity or behaviour. Avoidance of outdoor activity especially during twilight periods can limit the exposure to malaria transmission (Grambsch and Menne, 2003).

Certain cultural traits or social institutions function to limit malaria prevalence and mortality (Brown, 1981). This study indicated that cultural practices and taboos (which were not initially aimed at malaria prevention) such as avoiding movement around the village in the evening time, particularly by pregnant women, eventually became effective in reducing transmission. These traits do not exclusively function to limit malaria. Thus they had positive spill over effects over malaria prevention, or can be viewed as appropriated adaptation strategies. Based on the framework guiding this study, cultural practices as previously observed are adaptable to the prevailing conditions. Brown (1981) revealed that basic features of Sardinian culture had adaptive value against malaria endemicity. The settlement pattern, pastoral transhumance, and restricted mobility for pregnant women played a role in malaria prevention for the Sardinian community. Restrictions on mobility were more rigorous during pregnancy, which is perceived as a dangerous and delicate state in general, and which also presents more risk of malaria transmission. It is, therefore, sensible to postulate that low malaria prevalence for women is due to cultural rules restricting their mobility. In sum, these cultural traits reduce exposure to mosquitoes and consequently limit malaria rates among certain social groups (Brown, 1981).

Health workshops have been observed to also play a key role in adaptation to malaria endemicity in the Okavango Delta. The information disseminated in health workshops provide the people with the capacity to adapt to malaria incidence. Workshops are organised to raise community awareness among the locals through information sharing about climate change impacts on human health (Gonzales *et al.*, 2014). In the long-run, health workshops might build adaptive capacity to malaria endemicity in the study area through community education campaigns, especially that most respondents attend the workshops Capacity building is an essential step in adaptation process in all fields, including public health (Grambsch and Menne, 2003). This allows collaboration with the local stakeholders to restrain the spread of diseases that are sensitive to climatic variability Thus, education and awareness raising creates an environment that enables people to take well-informed, long-term, sustainable decisions (McMichael *et al.*, 2003).

Certain livelihood activities expose communities to risks that can accelerate the exposure and transmission of infectious diseases. They thus play an important role in occurrence of these diseases including malaria (Mphande, 2016). Diversifying livelihoods could be one way of reducing the risk of malaria transmission. In the Okavango Delta, households recognised diversification as a way of reducing malaria transmission risk. They diversified into small-scale businesses such as rural telephony activities, pasteries, and cigarettes. Depending on more than one source of income and food implies spending less time on certain activities (such as night fishing and irrigation) that present more risk of malaria transmission. This was also observed in Rusinga Island in Kenya where most people engaged in different occupations, some of which provided them food and money;they shifted from exclusive subsistence agriculture towards monetary or income-earning activities (Weckenbrock and Oldesloe, 2004). It has been observed that households tend to diversify livelihood strategies during challenging periods in order to adapt to change and reduce vulnerability (Ellis, 1999). Ultimately, having members of a household engaged in different livelihoods activities varies their level of exposure to risks (Mphande, 2016).

Cooking indoors as a strategy for wading off mosquito bites had the lowest rating on the scale of effectiveness because it has been proven that burning of woods indoors might not contribute significantly to the reduction in mosquito bites (Bockarie *et al.*, 1994). The potential use of alternative host species (such as livestock) to divert malaria vectors away from people has long been recognized as a potential environmental strategy for the reduction of malaria transmission (Bockarie *et al.*, 1994). Almost all the households in the Okavango Delta did not have livestock kraals in close proximity to homesteads. This was also the least effective strategy in protecting people against mosquito bites. Thus having livestock close to homesteads was not very well observed or recognised as a potential way of keeping the vector away from humans in the study area. A study conducted in Nigeria by Yakubu and Singh (2008) identified this strategy among other preventive measures andalso observed a higher mosquito population density inside cattle sheds than inside houses or outdoor resting boxes. However, this scenario might present a double whammy because it has been revealed that increased availability of alternative hosts (such as goats) could also enhance human malaria exposure, particularly to *Anopheles arabiensis*. This occurs when the heat and odour produced by animals attract a high number of vectors to the households which are close to where the animals are kept (Iwashita *et al.*, 2014).

## Conclusion

This study demonstrated that effective adaptation to malaria requires that individuals would need the capacity to recognise and respond to health threats. Health workshops have been found to be a base for capacity building against malaria endemicity, as they create a platform for information sharing on disease dynamics including prevention and vector behaviour. The study also showed that most households adhere to cultural practices (taboos) that restricted movement at certain times, particularly for pregnant women. Restricting activities to certain times of the day could help to reduce the risk of malaria transmission. Agricultural activities that happen at twilight periods underestimate or overlook the benefits derived from avoiding activities associated with mosquito bites. Therefore, limiting outdoor activity especially during twilight periods can reduce exposure to malaria transmission. Besides modern housing, poor communities such those in the Okavango Delta which cannot afford modern structures can benefit from modifications such as screening/installation of gauzes on their traditional houses. This strategy has been shown to contribute towards reduction in malaria transmission cases in other parts of the world. Although house screening has been shown to contribute towards reduction in malaria transmission cases in other parts of the world, the households in the study area have not realised the potential benefits of the strategy against malaria transmission. It is noteworthy that placing exclusive emphases on people’s potential contributions to eradicating malaria infection might unearth people-oriented, novel adaptation efforts in the end.

### Study limitations

The flood level had receded during data collection phase, and most respondents were harvesting weed and grass, gathering fruits, mostly mokuchomo/jackal berry (*Diospyros mespiliformis*) by the river. Therefore, most of them were not home during the day; they only withdrew from the above-mentioned activities in the evenings.

**List of abbreviations:**
CSO: Central Statistics Office
FGDs: Focus Group Discussions
IEC: Information Education and Communication
IRS: Indoor Residual Spraying
LLNs: Long-lasting Insecticidal Nets
MoHW: Ministry of Health and Wellness
NDHMT: Ngamiland District Health Management Team
NGOs: Non Governmental Organizations
SPPS: Statistical Package for the Social Sciences
SSA: Sub-Saharan Africa
UNDP: United Nations Development Programme
WHO: World Health Organization
